# Trends in Heroin Treatment Admissions in the United States by Race, Sex, and Age

**DOI:** 10.1001/jamanetworkopen.2020.36640

**Published:** 2021-02-05

**Authors:** Elodie C. Warren, Andrew Kolodny

**Affiliations:** 1 New York City Department of Health and Mental Hygiene, Bureau of Alcohol and Drug Use Prevention, Care and Treatment, Long Island City, New York; 2 Mailman School of Public Health, Columbia University, New York, New York; 3 Heller School for Social Policy and Management, Brandeis University, Waltham, Massachusetts

## Abstract

This cross-sectional study examines trends in heroin treatment admission rates in the United States by race, sex, and age from 2000 to 2017.

## Introduction

Over the past 25 years, the United States experienced a sharp increase in opioid use disorder (OUD) that disproportionately impacted White individuals in nonurban areas.^1^ In contrast, the heroin epidemic of the 1970s disproportionately impacted non-White individuals in urban communities, where prevalence of OUD remains elevated.^2,3^ Despite a decline in opioid-related mortality among White individuals in the US in 2018, overdose deaths continued to increase among Black individuals in the US.^4^ This suggests that efforts to address the crisis may not be benefitting Black communities. Historically, policy responses to drug problems in minority communities have focused on law enforcement rather than treatment. To better understand the epidemiology of OUD, we performed a cross-sectional analysis to examine heroin treatment admissions by race, age, and sex from 2000 to 2017.

## Methods

This cross-sectional study was approved by the Columbia University institutional review board. The research consisted of a secondary analysis of publicly-available, deidentified data, so patient informed consent was waived, per 45 CFR §46. The authors followed the Strengthening the Reporting of Observational Studies in Epidemiology (STROBE) reporting guideline.

The heroin treatment admission rates at state-regulated programs were examined for White, non-Hispanic individuals and Black, non-Hispanic individuals in 3-year groupings using the Substance Abuse and Mental Health Services Administration’s Treatment Episode Data Set (TEDS). Mean numbers of admissions per 100 000 in 3-year increments were calculated by age over time from 2000 to 2017 in the US. Denominators were obtained using the National Center for Health Statistics’ Bridged-Race Postcensal and Population Estimates. Final data analysis was performed using R statistical software version 3.6.3 (R Project for Statistical Computing) in December 2020.

## Results

**Figure 1.**
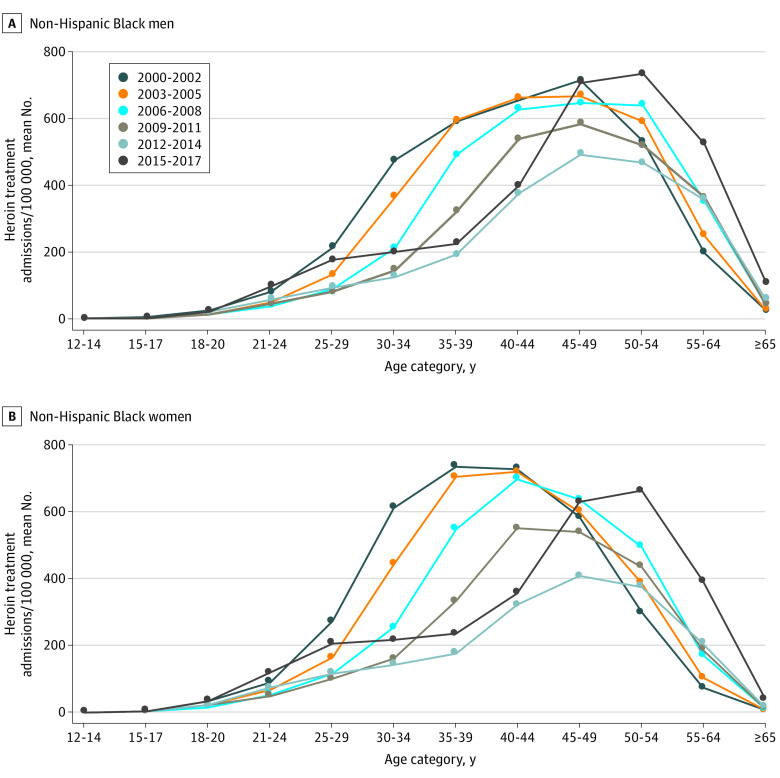
Heroin Treatment Admission Rates by Age Category Among Non-Hispanic Black Individuals, US, 2000-2017

**Figure 2.**
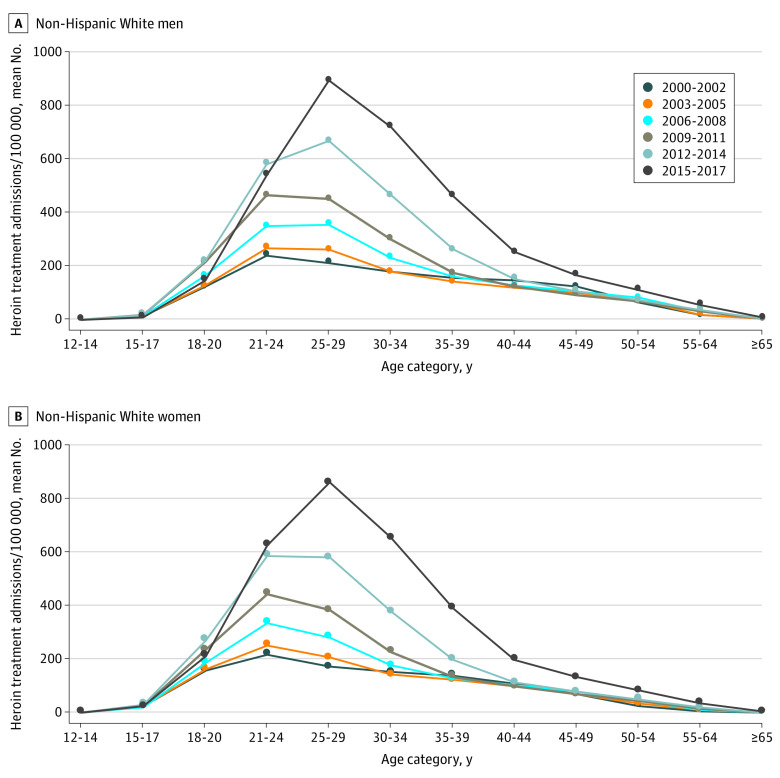
Heroin Treatment Admission Rates by Age Category Among Non-Hispanic White Individuals, US, 2000-2017

This study included a total of 6 087 697 patients admitted to state-regulated programs for heroin treatment between 2000 and 2017. Of these patients, 4 043 827 (66.4%) were male, 3 454 705 (56.7%) were White non-Hispanic, 1 055 059 (17.3%) were Black non-Hispanic, and 5 002 350 (82.2%) were aged 21 to 49 years. Admissions were higher among men than among women for both of the studied racial groups. Overall, White patients were younger than Black patients. Among White patients, heroin treatment admissions increased from 2000 to 2014; however, over the same time frame, there was an overall decrease in admissions for Black patients (Figure 1). Between 2014 and 2017, all groups experienced a large increase in admissions. Among White patients, the age distribution remained fairly constant (21-34 years) (Figure 2); yet among Black patients, it shifted toward older ages (concentrated at 45-54 years between 2015-2017) (Figure 1).

## Discussion

This study’s results suggest important differences in heroin treatment admissions to state-regulated programs by race and age in the US. Between 2015 and 2017, heroin treatment admissions among White men and women were highest for ages 21 to 34 years, whereas admissions among Black men and women were highest for ages 45 to 54 years. Among White individuals, the mean number of admissions per 3-year groupings increased since 2000 and the age distribution remained centered around ages 21 to 34 years. Among Black individuals, however, admissions decreased from 2000 to 2014 and the age distribution shifted to older ages. These results suggest the existence of an aging cohort of Black men and women seeking treatment for heroin addiction who developed OUD earlier in life. The reason for the recent rise in admissions in all groups may reflect improvements in access to care resulting from increased federal funding for addiction treatment.

Our results suggest a distinct epidemiology of OUD. In particular, it appears that a substantial population of older Black individuals continues to require OUD treatment. These population differences are in line with evidence that overdose deaths involving illicit opioids have disproportionately impacted non-White populations in Washington, DC, Baltimore, and other urban communities harmed by the heroin epidemic of the 1970s.^5^

This study has some limitations. As TEDS only captures admissions to state-regulated programs, admissions to private treatment programs and outpatient private practice settings may be underrepresented.^6^ Additionally, TEDS has been shown to overrepresent non-Hispanic Black individuals, and may underrepresent older adults. More research is needed to examine these limitations.

The failure to consider important population differences in efforts to address the opioid crisis may partially explain why opioid mortality in Black individuals in the US continued to increase in 2018 despite a decrease among White individuals. Policymakers should ensure adequate accessibility of evidence-based OUD treatment for the next several decades, with special attention paid to the needs of historically underserved communities. Future work should study opioid-related morbidity in other underserved groups.
