# Errors in Author Affiliation and Corresponding Author

**DOI:** 10.1001/jamanetworkopen.2021.2981

**Published:** 2021-02-25

In the Research Letter titled “Trends in Heroin Treatment Admissions in the United States by Race, Sex, and Age,”^1^ published February 5, 2021, an author affiliation was incorrectly included in 2 parts of the end matter. The New York City Department of Health and Mental Hygiene, Bureau of Alcohol and Drug Use Prevention, Care and Treatment affiliation should not have been included for Ms Warren. In addition, Dr Kolodny should have been named as the corresponding author instead of Ms Warren. This article has been corrected.^1^
