# Errors in Figures

**DOI:** 10.1001/jamanetworkopen.2021.10124

**Published:** 2021-04-20

In the Research Letter titled “Trends in Heroin Treatment Admissions in the United States by Race, Sex, and Age,”^1^ published February 5, 2021, panel B in both Figure 1 and Figure 2 were mistakenly given the same y-axis scales as panel A. The y-axis scale in Figure 1, panel B should have been labeled 0 to 400. The y-axis scale in Figure 2, panel B should have been labeled 0 to 700. This article has been corrected.^1^
